# Zebrafish Establish Female Germ Cell Identity by Advancing Cell Proliferation and Meiosis

**DOI:** 10.3389/fcell.2022.866267

**Published:** 2022-04-04

**Authors:** You-Jiun Pan, Sok-Keng Tong, Chen-wei Hsu, Jui-Hsia Weng, Bon-chu Chung

**Affiliations:** ^1^ Institute of Molecular Biology, Academia Sinica, Taipei, Taiwan; ^2^ Institue of Biochemistry and Molecular Biology, National Yang Ming Chiao Tung University, Taipei, Taiwan; ^3^ Institute of Biological Chemistry, Academia Sinica, Taipei, Taiwan

**Keywords:** gonad, transcriptome, ovary, oocyte, development, sex, sycp3, meiosis

## Abstract

Zebrafish is a popular research model; but its mechanism of sex determination is unclear and the sex of juvenile fish cannot be distinguished. To obtain fish with defined sex, we crossed domesticated zebrafish with the Nadia strain that has a female-dominant W segment. These fish were placed on a *ziwi:GFP* background to facilitate sorting of fluorescent germ cells for transcriptomic analysis. We analyzed the transcriptomes of germ cells at 10–14 days postfertilization (dpf), when sex dimorphic changes started to appear. Gene ontology showed that genes upregulated in the 10-dpf presumptive females are involved in cell cycles. This correlates with our detection of increased germ cell numbers and proliferation. We also detected upregulation of meiotic genes in the presumptive females at 14 dpf. Disruption of a meiotic gene, *sycp3*, resulted in sex reversal to infertile males. The germ cells of *sycp3* mutants could not reach diplotene and underwent apoptosis. Preventing apoptosis by disrupting *tp53* restored female characteristics in *sycp3* mutants, demonstrating that adequate germ cells are required for female development. Thus, our transcriptome and gene mutation demonstrate that initial germ cell proliferation followed by meiosis is the hallmark of female differentiation in zebrafish.

## Introduction

Zebrafish is a popular animal model for the studies of development, toxicology, gene regulation, and biomedicine. Zebrafish facilities need to maintain enough fish of both sexes to ensure normal production of offspring. Yet zebrafish sex ratio is often skewed by stress caused by environmental factors such as temperature, density, food intake, and hormones (Santos et al., 2017). For example, zebrafish become male-biased when temperatures are too high (Uchida et al., 2004) or too low (Sfakianakis 2012). Fish reared at high population density tend to become males (Liew et al., 2012; Sfakianakis 2012; Ribas et al., 2017). Treatment with 17β-estradiol causes fish feminization (Andersen et al., 2001). Higher food intake also promotes female development (Lawrence et al., 2008).

In addition to environmental factors, genes also control zebrafish sex differentiation. Zebrafish belongs to the ZZ/ZW sex-determining system, in which female genes play a dominant role in sex determination (Tong et al., 2010; Wilson et al., 2014). The sex of juvenile zebrafish cannot be distinguished until the appearance of secondary characteristics after about 3 months of age. In addition, domesticated zebrafish strains do not have discernable sex chromosomes (Anderson et al., 2012). However, wildtype Nadia zebrafish have a W-sex locus located at the tip of chromosome 4 (Anderson et al., 2012). This region contains a sex-associated region in ZW females. Although ZZ fish that lack the W locus are always males, the ZW fish are not always females (Anderson et al., 2012). A small proportion of the ZW fish can become males depending on the rearing condition, further complicating the study.

The domesticated zebrafish strains do not have the W locus; and their sex differentiation can be affected by many genes (Liew et al., 2012). Depletion of *follicle-stimulating hormone receptor* leads to sex reversal to males due to the failure of follicle maturation (Zhang et al., 2015). Mutation of *gonadotropin-releasing hormone* 3 (*Gnrh3*) leads to male-biased sex ratio (Feng et al., 2020). In addition, zebrafish with a null mutation of *tudor domain-related proteins* (*Tdrds*) also become infertile males (Dai et al., 2017).

Sex steroids control the differentiation of sex organs. Cyp19a1a catalyzes the synthesis of estrogen; its mutation leads to the development of only male zebrafish because of the lack of estrogen production (Lau et al., 2016). Cyp17a1 catalyzes the synthesis of all sex steroids; its mutation eliminates ovary but retain testis development, indicating that zebrafish gonad development can tolerate androgen deficiency more than estrogen deficiency (Zhai et al., 2018). Cyp11a2 catalyzes the synthesis of all steroids; its depletion causes testis enlargement and feminized secondary sexual characteristics (Wang et al., 2022). Cyp11c1 controls the synthesis of cortisol and 11-ketotestosterone; its defect does not alter sex determination, but leads to defect in oocyte maturation and testis function (Zhang et al., 2020).

Zebrafish sex differentiation is affected by the number of germ cells. During zebrafish sex differentiation, testes often form from juvenile ovary via oocyte apoptosis (Uchida et al., 2002). Germ cell ablation leads to the development of male fish (Slanchev et al., 2005). Mutations of genes such genes as *vasa* (Hartung et al., 2014), *protein arginine methyltransferase 5* (Zhu et al., 2019), and *fancl* (Rodriguez-Mari et al., 2010) all lead to germ cell loss, and result in the development of male fish in the adult. On the contrary, fish become females if their larvae are induced to produce more germ cells (Ye et al., 2019). These results indicate that germ cell number is an important factor that controls the direction of zebrafish sexual differentiation. It is still unclear how germ cells interact with various genes leading to final sexual characteristics.

In this article, we analyzed events leading to the first step of sexual differentiation. We designed a method to differentiate the sex of juvenile fish based on the presence of the W locus, and performed transcriptomic analysis to identify genes differentially expressed in the presumptive males and females at 10, 12, and 14 days postfertilization (dpf). We found that differential cell proliferation and meiosis are the earliest signs of zebrafish sex differentiation. We also generated mutants deficient in *synaptonemal complex protein 3* (*sycp3*); these mutants became exclusively sterile males due to meiotic arrest followed by germ cell apoptosis. Thus, our study delineates the importance of germ cell proliferation and meiosis in the initial female sex differentiation of zebrafish.

## Materials and Methods

### Zebrafish Strain

The *Tüpfel long fin* (TL) and Nadia strains of zebrafish (*Danio rerio*) were kept at 28.5°C, pH7.0, following the guidelines of Institutional Animal Care and Utilization Committee of Academia Sinica. The *tp53* ^*zdf1*^ fish were obtained from Zebrafish International Resource Center (Berghmans et al., 2005).

The *Tg(ziwi:GFP)* transgenic fish were generated after injecting the *Tol2 transposase* mRNA (synthesized from *pCS2FA-transposase*) and the plasmid that contains a Tol2 cassette harboring a *GFP* cDNA with an SV40 poly(A) sequence at the 3′ and the 4.8-kb *ziwi* promoter (−4690 to +135) at the 5′ (Leu and Draper 2010). This plasmid was generated by the Gateway recombination-based cloning as described previously (Kawakami 2005; Villefranc et al., 2007; Leu and Draper 2010).

The W- and Z-hybrid were generated by mating Nadia (NA) with *Tg(ziwi:GFP)* fish in TL background. Around 30% of Nadia ZW females reversed their sex to become adult male fish (ZW^SR^) in our fish facility. These ZW^SR^ males were crossed with ZW females and their offspring would be predicted to be 25%ZZ, 50%ZW, and 25%WW fish. We screened these females (ZW and WW) for the ones that only gave rise to offsprings with the W locus. These females were labeled as the WW females. Next, we used the WW outcross with *Tg(ziwi:GFP)* to get the W-hybrid. We also mated ZZ Nadia fish with *Tg(ziwi:GFP)* fish (in TL background) to obtain Z-hybrid.

The CRISPR/Cas9 approach was used to generate the *sycp3* mutant fish. The sgRNA target sequences located on *sycp3* exon2 were selected using CHOPCHOP and Benchling online program (Supplementary Table S1). We injected 40 pg/nl (final concentration) of the *sycp3* single guide RNA and 250 pg/nl (final concentration) Cas9 protein simultaneously into one-cell stage embryo. Examination of genotypes of 10 founder fish embryos indicated that knockout efficiency was 76%. The founders (F0) were outcrossed with WT to get the offspring (F1), which were genotyped at 3 months, and the sites of mutation confirmed by sequence analysis. F1 fish lines were maintained for further use.

### PCR and *in vitro* Transcription

The PCR primers are listed in Supplementary Table S1. The PCR primers were annealed, extended by 3 cycles of PCR reactions (95°C 2 min, 50°C 2 min, and 72°C 2 min) and stored at 10°C. The sgRNA primers were designed to contain a T7 promoter and overlap with Cas9 guide-constant sequence (Talbot and Amacher 2014). After the PCR fragment was purified by a QIAGEN PCR clean kit, the sgRNA was generated by *in vitro* transcription at 37°C for 2 h using a MEGAshortscript T7 Kit (AM1354, Thermo Scientific). After digestion of dsDNA template by 1 μl Turbo DNase 37°C at 15 min, sgRNA was precipitated and purified.

### Fin Clip for Genotyping

Juvenile fish were anesthetized in tricaine solution before fin clipping. The fin was lysed in 50 mM NaOH 20 μl, and incubated at 95°C for 15 min to release its genomic DNA, which was then neutralized by the addition of 1 mM Tris pH8.0 (2 μl) and centrifuged for 1 min. The genomic DNA in the supernatant was used for PCR amplification. PCR fragments were analyzed by capillary electrophoresis using a Qiaxcel DNA screening kit.

### Fish Fertilization Test

Male *sycp3* ^*as8d/as8d*^ and fish were crossed with wildtype females for three practice mating before the fertilization test. The fertilization rate was scored as the number of successful egg laying followed by normal development to the dome stage, 70% epiboly and 24 hpf.

### Fish Staging, Gonad Dissection, and Dissociation for Germ Cell Sorting

The criteria for fish staging are stringent; only fish that were 5.2 mm at 10 dpf, 5.7 mm at 12 dpf, and 6.2 mm at 14 dpf were used. The larvae were dissected in phosphate buffered saline (PBS) by forceps for the removal of head, intestine and tail. The dissection took less than 30 min to ensure germ cell viability for successful sorting. Gonadal cells from 25 to 50 larvae were dissociated with 0.25% trypsin and 0.2% collagenase for 30 min at 28°C before enzyme inactivation in 100% fetal bovine serum. The dissociated cells were filtered through a 30-nm strainer to remove tissue remnants. Then, GFP^+^ germ cells were collected in 96 wells by FACS instrument Aria II (BD biosciences). The 100-µm nozzle was used in the FACS sorter, and allowing approximately 15–20 min for the sorting of each sample. Around 300–500 germ cells were obtained from the 10-dpf gonads, 500–700 germ cells from 12-dpf gonads, and 1000 germ cells from 14-dpf gonads. Germ cells were lysed inside the 96-well plate and stored at −80°C.

### cDNA Preparation and Transcriptome

Two different methods were used to prepare cDNA libraries as summarized in Supplemental Table S2. Initially, 12- and 14-dpf cDNA libraries were prepared following the Quartz-seq procedure (Sasagawa et al., 2013). Later, more batches of 10- and 12-dpf cDNA libraries were prepared using the Smart-seq HT procedure (Takara bio). In SMART-seq HT, full-length cDNA was synthesized in one step, and then amplified by 12 cycles of PCR. After purification with the AMPure XP kit (Beckman Coulter), cDNAs were analyzed by a bioanalyzer for their qualities. We used 150 pg of cDNAs as input to prepare libraries using Illumina Nextera XT DNA Library Preparation Kit. The concentrations and lengths of cDNAs in the libraries were about 10 nM and 200 bps, respectively. Sequences of the cDNA libraries were determined by reading 75-bp paired ends (Illumina NextSeq 500 2*75 bps, v2). The resulting sequences from all experiments were first presented as transcript per million (TPM), then normalized and filtered by the method of trimmed mean of M values (TMM) using the edgeR package. The zebrafish genome GRCz11 was used as a reference genome.

### Real-Time Quantitative PCR

The real-time quantitative PCR was performed using a kit (QuantiFast SYBR Green RT-PCR Kit, QIAGEN) with sample dilution for the standard curve in a Roach LightCycler480. The PCR condition was 95°C 5 min and 60°C 30 s and 72°C 5 min for 55 cycles. The RNA level was normalized using the *ef1a* level as an internal control.

### Whole-Mount Fluorescent *In situ* Hybridization and Immunofluorescence Staining

Larval samples were fixed with 4% PFA overnight at 4°C before dissection. Whole-mount fluorescent *in situ* hybridization was performed following a previously published protocol (Brend and Holley 2009). For immunofluorescence staining, after whole-mount fluorescent *in situ* hybridization and PBST (0.1% Triton X-100 in PBS) wash, samples were incubated with 10% normal goat serum at R.T. for 3 h, then with primary antibody at 4°C overnight, followed by incubation with secondary antibody (diluted 1:500) and DAPI at R.T 1 h.

For double immunostaining, samples were incubated with mouse anti-proliferating cell nuclear antigen (PCNA) and rabbit anti-Vasa antibodies simultaneously at 4°C overnight before incubation with secondary antibodies (anti-mouse Alexa fluor 546 and anti-rabbit Alexa fluor 488). The antibody information is shown in Supplementary Table S1. For Sycp3 and Vasa double staining, we first labeled Vasa antibody with 633 DyLight Dye using the DyLight Microscale Antibody Labeling Kit (Thermo) for later use. Samples were incubated with rabbit Sycp3 antibody at 4°C overnight, followed by incubation with Alexa fluor 488-conjugated secondary anti-rabbit antibody at R.T 1 h. Next, samples were washed with PBST before incubation with DyLight 633-conjugated Vasa antibody at 4°C overnight. Samples were washed and the gonads were dissected for confocal imaging on the following day.

### Counting of Germ Cell Numbers

Germ cells were visualized by fluorescent vasa staining before manual counting under a ZEISS LSM 710 confocal microscope equipped with ZEN 2009 (Black edition) software. Germ cells in all Z sections were scored.

### Microscopy and Photography

The images of immunofluorescence for Sycp3, Rad51, Casp-3 were analyzed by a ZEISS LSM 710 confocal microscope equipped with ZEN 2009 (Black edition) software.

### Statistics Analysis

The data are shown as mean ± standard deviation. The unpaired two-tailed Student’s *t*-test was used for two-group comparisons. Chi-squared analysis was used for comparing meiotic progression.

### Bioinformatics Analysis

The RNA-sequencing results were analyzed by Gene Ontology from Metascape website (https://metascape.org), Venn diagram from Venny website (https://bioinfogp.cnb.csic.es/tools/venny/), MA plot, volcano plot, Z-score analyzed in Prism. All RNA-seq data files are available from the Gene Expression Ominbus database (accession number GSE196899).

## Results

### Establishment of the Method for Differentiating Presumptive Female and Male Germ Cells

**FIGURE 1:**
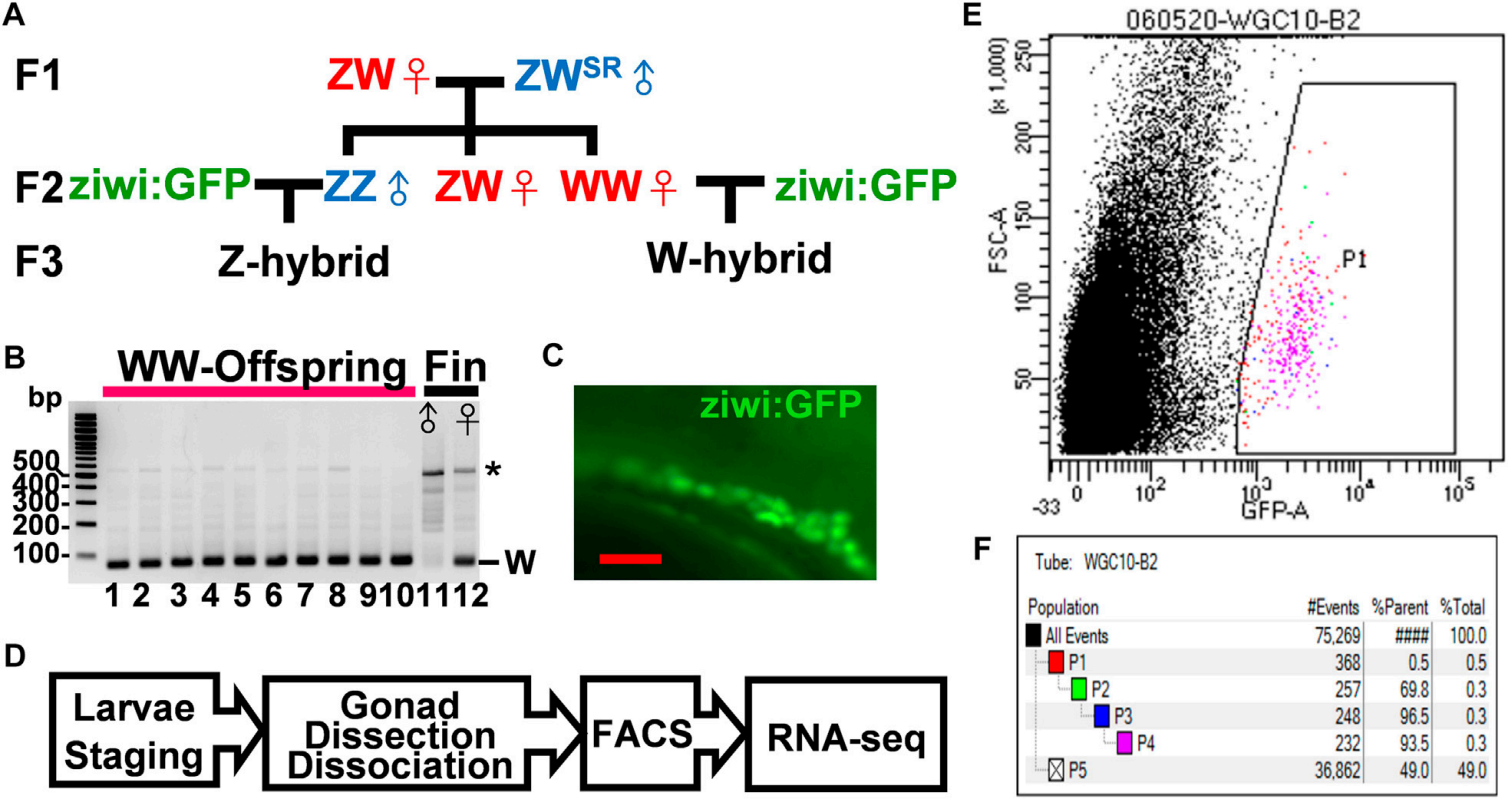
Isolation of germ cells from juvenile zebrafish with defined sex. **(A)**. The flow chart for the generation of W- and Z-hybrids. Sex-reversed ZW^SR^ Nadia males were crossed with ZW females to obtain the WW females in the F2 generation. The WW and ZZ fish were each mated with the Tg (ziwi: GFP) in the TL genetic background to obtain W- or Z-hybrid offspring with fluorescent germ cells. **(B)**. Gel electrophoresis showing the detection of the W-fragment in the genome of 10 offsprings from the WW mother after PCR amplification (lanes 1–10). Lanes 11 and 12 are genomic DNAs from the fin of a male and a female of the Nadia strain as controls, respectively. The W fragment is 86 bps in length. The star marks nonspecific PCR bands. **(C)**. The gonad of a Tg (ziwi: GFP) fish with fluorescent germ cells. **(D)**. The flow chart for transcriptome analysis. Larvae were first staged to ensure proper development before their gonads were dissected. Their germ cells were then dissociated and sorted by FACS before RNA-seq analysis. **(E)**. A representative FACS plot shows the selection of GFP-positive germ cells (P1). **(F)**. A typical FACS data set showing the procurement of 232 GFP^+^ germ cells (pink group, 0.3% of all cells). Blue-group (P3) and pink-group (P4) cells were obtained after cell size quantity control. The green group (P2) selects for live cells that do not stain with propidium iodide. Black dots represent other cells and cell debris.

A big hurdle in the investigation of zebrafish sexual development is the difficulty to identify the sex of juvenile fish. Because the laboratory strains lack sex chromosomes, we turned to wildtype Nadia strain (NA), which has a W locus in females only (Wilson et al., 2014). We designed a scheme to obtain fluorescent germ cells from gonads with defined sex (Figure 1A). In this scheme, we selected Nadia fish of the ZW genotype that was naturally sex reversed to males. We mated these ZW males with ZW females, and screened the offspring for WW females, which invariably produced all ZW female offspring as shown by the presence of the W fragment in PCR analysis (Figure 1B). The control DNA contained the W fragment only in the female but not in the male fin (Figure 1B). The WW fish were crossed with transgenic *Tg(ziwi:GFP)* fish in a genetic background of *Tüpfel long fin* (TL), which express GFP in all germ cells (Figure 1C). All offspring from this mating contained the W-locus in a genetic background of Nadia/TL hybrid. These W-hybrid were presumptively all females and a half of them had fluorescent germ cells. With the same scheme, we mated the ZZ Nadia father with the Tg (ziwi:GFP) mother to obtain the Z-hybrid that was presumptively all males (Figure 1A). The fluorescent germ cells of both W and Z hybrids could be sorted by fluorescence-activated cell sorter (Figure 1D).

Our earlier work indicates that germ cells start to become sex-dimorphic at 10–14 dpf (Tong et al., 2010). In order to get germ cells at 10–14 dpf, we staged about 20–50 larvae (6.2 mm at 14 dpf, 5.7 mm at 12 dpf, and 5.2 mm at 10 dpf), and sorted their germ cells using a fluorescence-activated cell sorter for bulk RNA-seq (Figure 1E). About 200–500 GFP^+^-germ cells (0.5% of total cells) were obtained from each preparation (Figure 1F). Two independent RNA-seq experiments each containing many batches of cDNAs were performed. In the first experiment, we prepared cDNAs from 12- and 14-dpf germ cells by Quartz-seq, and in the second one we prepared cDNAs from 10- and 12-dpf germ cells by Smart-seq (Supplementary Table S2). Besides, we also performed RNA-seq of trunk cells for comparison as a negative control. These samples were normalized by comparing the trimmed means of the M-value (TMM) (Supplementary Figure S1A), and their gene expression compared in Volcano Plots (Supplementary Figure S1B). With a screening criterion of 1.5-fold change, transcripts per million>5, and false discovery rate<0.05, 668 and 1690 differential expression genes (DEGs) were obtained from the 10- and 14-dpf samples, respectively (Supplementary Figure S2). The data from 12-dpf samples were the intermediate of the other stages, either similar to that of the 10-dpf sample or the 14-dpf sample, and therefore were examined for validation only.

### Identification of Meiotic Genes That Mark Presumptive Female Germ Cells at 14 dpf

**FIGURE 2:**
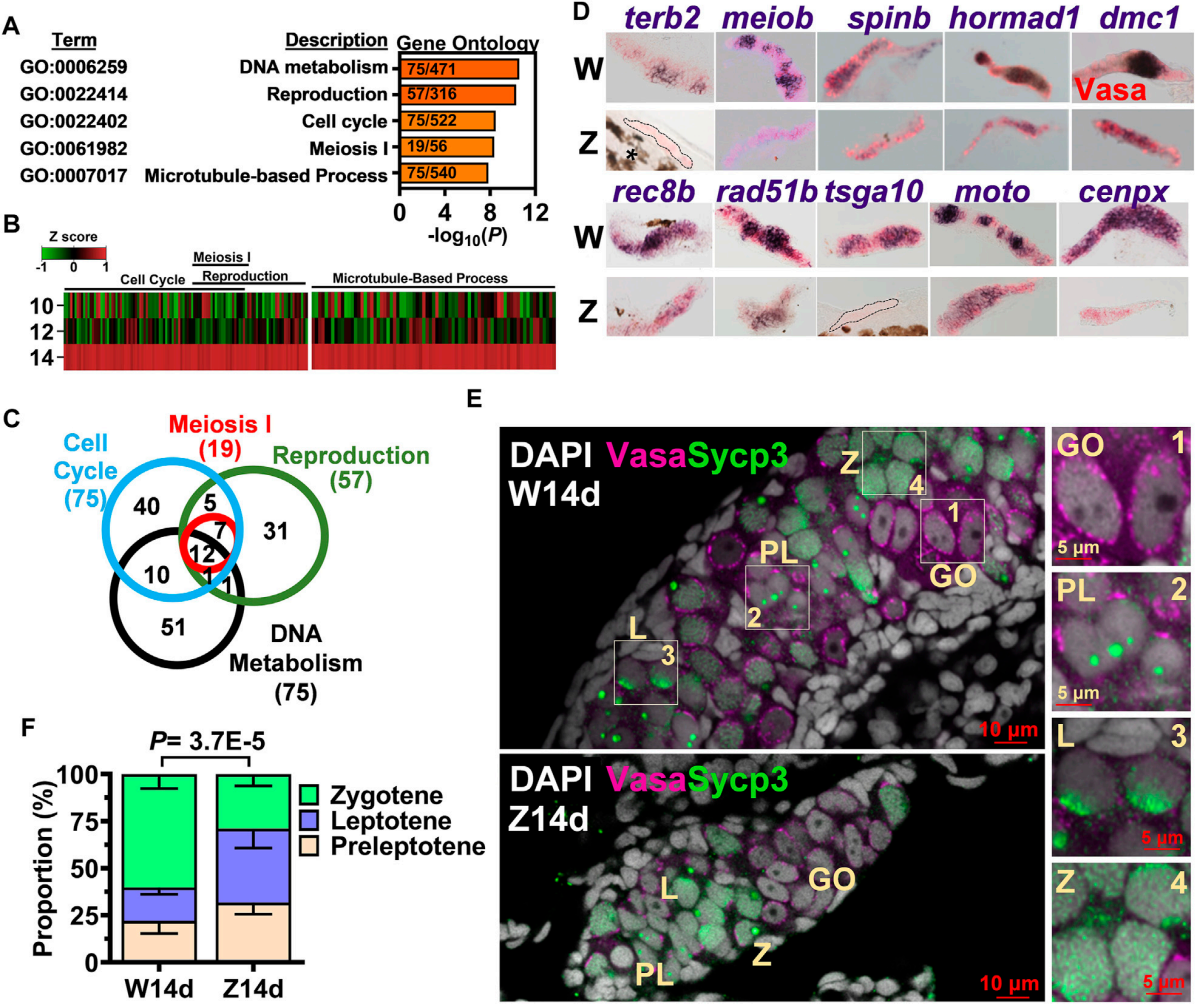
Meiosis as the hallmark of 14-dpf female germ cell transcriptome. **(A)**. Gene ontology analysis of 1692 genes upregulated in the W-hybrid. **(B)**. Expression of genes in the meiosis I, reproduction, cell cycle, and DNA metabolism at 10, 12, and 14 dpf. One gene is represented by a line, and the color represents the expression level normalized by Z-score. **(C)**. A Venn diagram showing the overlaps of genes involved in meiosis I, reproduction, cell cycle, and DNA metabolism. **(D)**. *in situ* hybridization of 10 meiotic genes in the 14-dpf W- and Z-hybrids. **(E)**. The immunostaining of Sycp3 in gonocyte (GO), preleptotene (PL), leptotene **(L)**, and zygotene (Z) germ cells. Germ cells marker Vasa and meiotic gene marker Sycp3 are magenta and green, respectively. DAPI are shown in white. The boxed regions are enlarged at the right side (1–4). **(F)**. The W-hybrid advances meiosis faster than the Z-hybrid at 14 dpf. The proportion of preleptotene (yellowish), leptotene (blue), and zygotene (light green) are shown. The *p* value was calculated using the chi-squared analysis.

From the 1692 genes upregulated in the 14-dpf W-hybrid, gene ontology analysis indicated that these genes were enriched in such processes as DNA metabolism (GO:0006259), reproduction (GO:0022414), cell cycle (GO:0022402), meiosis I (GO:0061982) and microtubule-base process (GO:0007017) (Figure 2A). All these genes were sporadically expressed at 10 and 12 dpf, then upregulated at 14 dpf (Figure 2B). A Venn diagram shows that these processes share similar gene sets. All 19 differentially expressed meiotic genes are included in the gene terms for reproduction and cell cycles (Figure 2C). This result indicates that meiosis-I has properties of reproduction, DNA metabolism, and cell cycles. We therefore focused on meiosis for further characterization.

We examined the expression of 10 randomly picked meiotic genes by *in situ* hybridization. The expression of these 10 genes, *dmc1, hornad1, spin, meiob, terb2, cenpx, moto, tsga10, rad51b, rec8b* were all higher in the W- than the Z-hybrid (Figure 2D), thus validating our RNA-seq data.

Germ cells from premeiotic gonocyte to the diplotene stage of meiotic prophase can be differentiated by their size and pattern of DAPI-staining (Elkouby et al., 2016). While 14-dpf gonocyte (GO) nuclei were evident with one or a few nucleoli, leptotene (L) germ cells (GC) contained condensed DAPI with multiple nucleoli (Figure 2E). The zygotene (Z) GCs had a big and round bouquet-like nucleolus. Synaptonemal complex protein 3 (Sycp3) staining was not apparent in the premeiotic gonocytes (Figure 2E, enlarged in 1), but started to appear in the nucleoli of preleptotene (PL) cells as small dots (Figure 2E, enlarged in 2), became a cap in the nuclear boundary of the leptotene cell (Figure 2E, enlarged in 3), and as a big dot in the bouquet nucleolus as well as a small dotted net in the nucleus of zygotene germ cells (Figure 2E, enlarged in 4). We compared the proportions of meiotic germ cells in 14-dpf germ cells of both W- and Z-hybrids, and found that the W-hybrid had faster meiotic progression with a higher proportion of zygotene germ cells (Figure 2F). These results indicate that a prominent feature of a 14-dpf presumptive female gonad is higher expression of meiotic genes and advanced meiotic progression.

### Disruption of *sycp3* Affects Gonad Differentiation and Leads to Meiotic Germ Cell Arrest in Zebrafish

**FIGURE 3:**
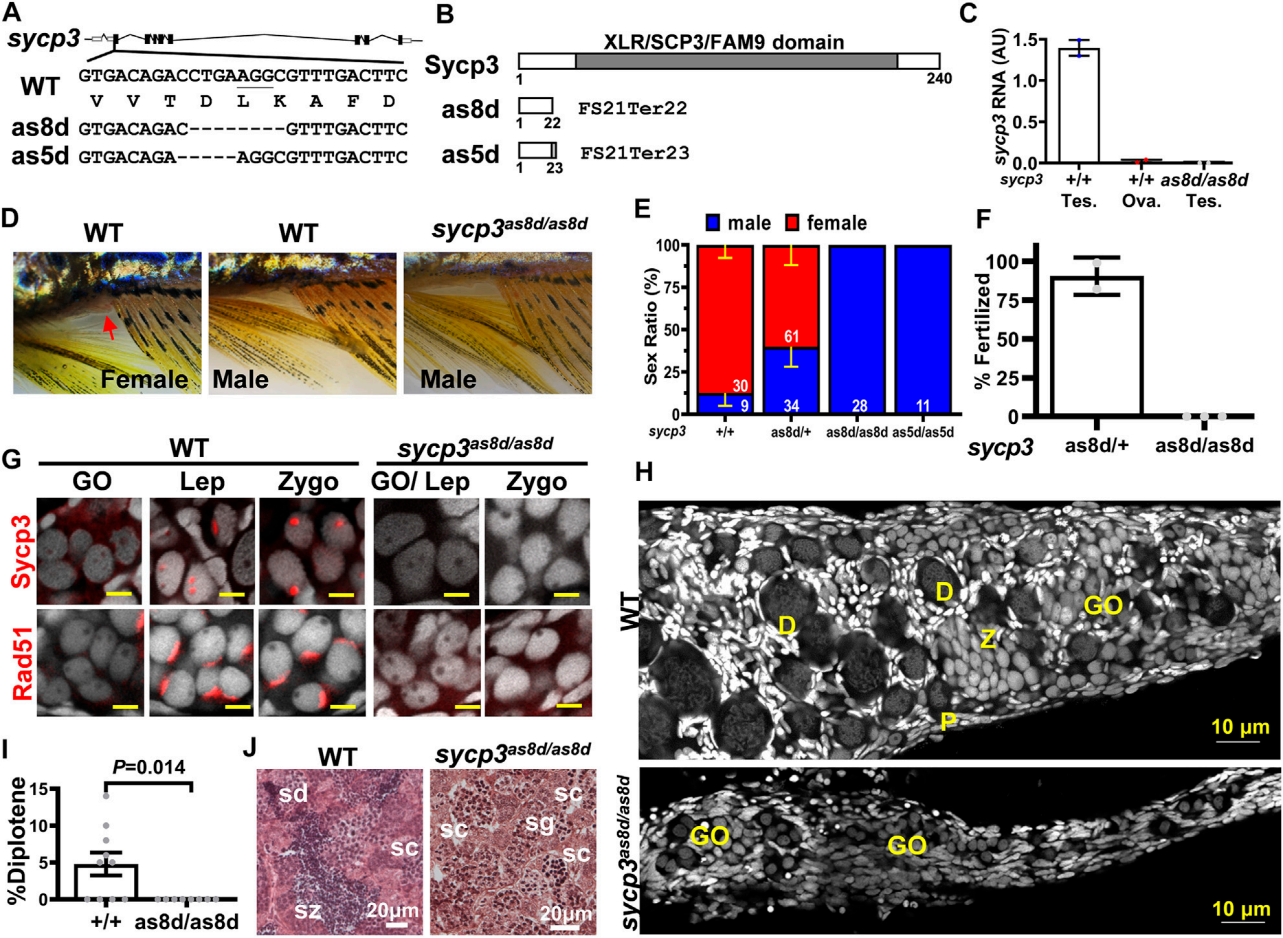
Disruption of sycp3 leads to infertile males with germ cell arrest during meiosis. **(A)**. Maps of *sycp3* and the sites of the mutations. The mutations are located in *sycp3* exon 2. **(B)**. The domain structures of wild type and mutant Sycp3. Both *as8d* and *as5d* mutations cause premature protein termination. **(C)**. The RNA expression of *sycp3* decreased in sycp3 mutant. **(D)**The external morphology of WT and *sycp3* mutants. Females have a genital papilla (arrowhead) and yellowish fins. Males lack genital papilla and their fins are brownish. **(E)**. The Sex Ratio of *sycp3* mutants. The male is represented by the blue bar. Red bar represents females. The numbers of fish used in the analysis are shown inside the bars. **(F)**. Fertilization test showing the frequency of 3-month male fish (genotype indicated below the *X*-axis) to produce offspring. **(G)**. Immunofluorescence staining of 14-dpf WT and *sycp3* ^*as8d/as8d*^ gonads with Sycp3 and Rad51 antibodies (Wilson, High et al.). DAPI staining is shown in white color. The developmental stages of the meiotic germ cells are marked at the top of each figure. GO: gonocyte, Lep: leptotene, Zygo: zygotene. **(H)**. The 1-month WT but not *sycp3* ^*as8d/as8d*^ gonad contain diplotene germ cells. D: diplotene, P: pachytene. **(I)**. Quantitation of the % diplotene germ cells in a gonad. The *p* values were calculated using Student’s *t* test. **(J)**. Testicular morphology showing *sycp3* ^*as8d/as8d*^ mutant germ cells do not go beyond spermatocytes (sc) while WT germ cells reach spermatozoa (sz). sg: spermatogonia, sd: spermatid.

In order to dissect the functions of early meiotic genes, we disrupted a meiotic gene, *sycp3* (Synaptonemal complex protein 3) by CRISPR/Cas9. Two *sycp3* mutant lines, *as8d* (deletion from +350 to +357) and *as5d* (deletion +349 to +353) were generated (Figure 3A). All these lines were predicted to result in early protein truncation and complete loss of gene functions (Figure 3B). *Sycp3* mRNA was expressed in the testis but not in the ovary in the WT adults (Figure 3C). It was also absent in the *sycp3* ^*as8/as8d*^ mutant testis, indicating a complete loss of *sycp3* mRNA expression as a result of the gene mutation. We examined the body weight (Supplementary Figure S2A), body length (Supplementary Figure S2B), and gonadosomatic index (%GSI) of *sycp3* mutants (Supplementary Figure S2C), and found they were all normal, indicating that these gene mutations did not affect zebrafish growth. But the *sycp3* mutant lines developed into only males, which lacked genital papilla and had the characteristics of brown fin color (Figure 3D). Both *sycp3* ^*as5d/as5d*^ and *sycp3* ^*as8d/as8d*^ lines were 100% males, (Figure 3E). These *sycp3* ^*as8/as8d*^ males were infertile in mating tests (Figure 3F).

We next examined meiotic germ cells by staining with DAPI and Sycp3 antibody. While Sycp3 was present in leptotene and zygotene germ cells of 14-dpf WT gonads, it was absent in the *sycp3* mutants (Figure 3G), demonstrating that these mutants were devoid of Sycp3. Rad51 recombinase was detected in the WT leptotene and zygotene cells as a cap, but it was absent in the *sycp3* mutant gonads (Figure 3G). This result indicates that *sycp3* mutation has pleiotropic effects affecting other proteins such as Rad51.

We examined the progression of meiosis in *sycp3* mutants at 1 month of age. Germ cells of all stages from gonocyte (GO), leptotene (L), pachytene (P), to diplotene (D) were present in the WT gonad (Figure 3H). But the *sycp3* ^*as8d/as8d*^ gonads did not contain diplotene (D) germ cells (Figures 3H,I). This result indicates that *sycp3* ^*as8d/as8d*^ germ cells cannot progress to the diplotene stage. At 2 months of age, the WT testis contained sperm cells of all developmental stages from spermatogonia to spermatozoa, but the *sycp3* ^*as8d/as8d*^ testis only contained germs cells up to the spermatocyte stage (Figure 3J). It indicates the *sycp3* mutation also causes meiotic arrest of male germ cells. Thus, the failure of zebrafish *sycp3* ^*as8d/as8d*^ germ cells to pass through the pachytene stage of meiosis leads to infertility and female-to-male sex reversal.

### Germ Cell Apoptosis Leads to Sex Reversal of *sycp3* Mutants

**FIGURE 4:**
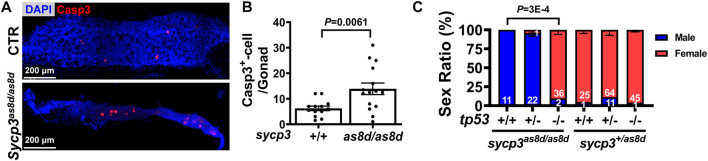
The sex reversal of sycp3 mutant fish is caused by germ cell apoptosis and can be compensated by additional tp53 mutation. **(A)**. The immunostaining of apoptotic marker Caspase-3 (Wilson, High et al.). DAPI staining is blue for nuclei. **(B)**. Quantification results of Caspase-3. Apoptotic germ cells are increased in the 1 month old *sycp3* ^*as8d/as8d*^ gonad. **(C)**. The sex ratios of *tp53* ^*-/*-^;*sycp3* ^*as8d/as8d*^ mutants. While *sycp3* ^*as8d/as8d*^ fish are all males, many females have the *tp53* ^*-/-*^ *;sycp3* ^*as8d/as8d*^ genotype. Blue bar represents males and the red bar represents females.

Zebrafish sex reversal is often caused by germ cell apoptosis (Rodriguez-Mari et al., 2010). To examine whether sex reversal in *sycp3* mutants is related to germ cell apoptosis, we stained 1-month gonad with a marker for apoptosis, cleaved Caspase 3 (Figure 4A). The number of Caspase-3^+^ GC was significantly higher in *sycp3* ^*as8d/as8d*^ mutant (Figure 4B), indicating increased cell death. Since *tp53* prevents cell apoptosis (Rodriguez-Mari et al., 2010), we crossed *tp53* mutant fish with *sycp3* mutants to prevent their cell apoptosis. While all the *sycp3* ^*as8d/as8d*^ mutants were males, many of *tp53* ^*-/-*^ *;sycp3* ^*as8d/as8d*^ double mutants were females Figure 4C. This result indicates that the prevention of cell apoptosis alleviates zebrafish sex reversal caused by *sycp3* deficiency. These results show that the sex reversal in *sycp3* mutants is caused by germ cell apoptosis.

### Identification of Cell Proliferation That Marks Presumptive Female Germ Cells at 10 dpf

**FIGURE 5:**
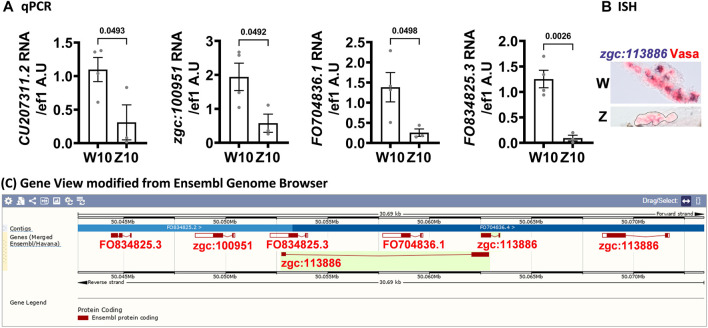
Genes clustered on chromosome 17 are upregulated in 10-dpf germ cells of the W-hybrid. **(A)**. Higher expression of CU207311.2, zgc:100951, FO704836.1, FO834825.3, in the W-hybrid than the Z-hybrid at 10 dpf as detected by RT-PCR. The *Y*-axis is the RNA level in arbitrary units (A.U.) after normalization with an internal control (*ef1*). **(B)**. The expression of *zgc:113886* (blue-purple) is higher in the W-hybrid shown by *in situ* hybridization. Vasa antibody stains germ cells red in Alexa fluor 546. **(C)**. All these upregulated genes are clustered in the same locus on chromosome 17 as shown by the Ensembl Gene view.

We examined genes that were upregulated in the W- versus Z-hybrid by RNA-seq at 10 dpf and validated some of top differentially expressed genes by qPCR (Figure 5A) and *in situ* hybridization (Figure 5B). Many of these genes are clustered in the same locus of chromosome 17 (Figure 5C), indicating they are probably coordinately regulated. Because these genes are all novel genes, their detailed functions still await further characterization. The only clue is that some of these genes (e.g., *FO704836.1, FO834825.3, zgc:112399*) were annotated to be involved in cell proliferation, while zgc:113886 and zgc:100951 were annotated to be C2H2-type zinc finger proteins that participate in pol2 transcription.

**FIGURE 6:**
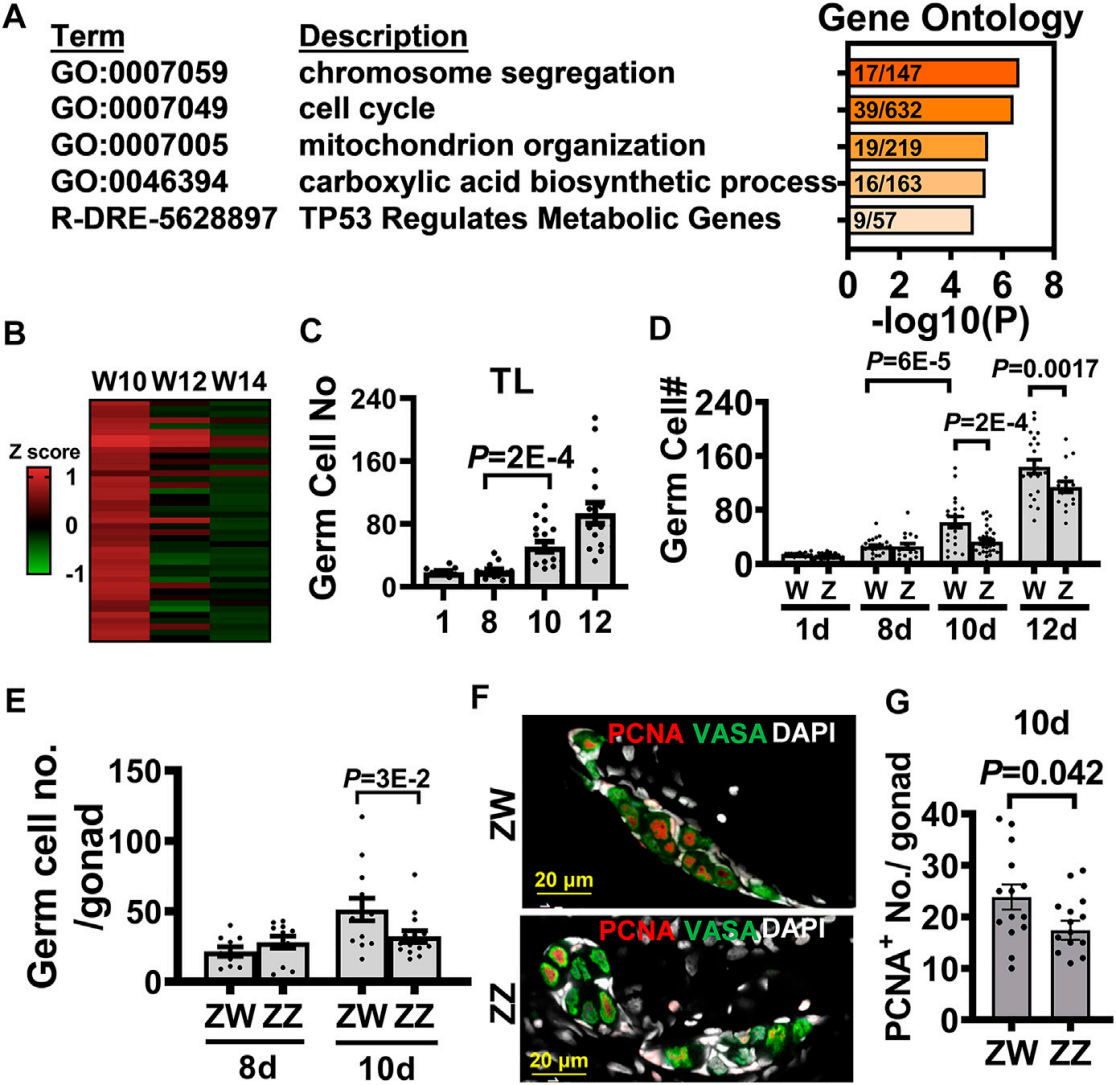
Transcriptome analysis of 10-dpf germ cells identifies the advanced germ cell proliferation as a sign of zebrafish female differentiation. **(A)**. Gene ontology analysis of 668 differentially expressed genes (DEGs) upregulated in the 10 dpf W-hybrid. **(B)**. The heat map of DEGs in the pathways of chromosome segregation (GO:0007059) and cell cycle (GO:0007049) are high in the W-hybrid at 10 dpf and gradually decreased at 12 and 14 dpf. The expression level is normalized by a z-score **(C)**. The germ cell numbers of TL laboratory strain at 1, 8, 10, and 12 dpf. Germ cells significantly proliferate at 10 dpf. **(D)**. The germ cell numbers of W- and Z-hybrid become different from 10 dpf on. **(E)**. Germ cell number in ZW genotype of Nadia is higher than that in ZZ gonad at 10 dpf. **(F)**. Double immunofluorescence showing PCNA staining for proliferating germ cell and Vasa (green) for germ cells. DAPI staining is white for nuclei. **(G)**. Quantitation of PCNA^+^-germ cells, showing ZW gonads contain more proliferating germ cells than ZZ gonads at 10 dpf. One dot represents data from one gonad. The two-tailed and unpaired student *t*-test used for statistical analysis.

Upon gene ontology analysis, the 668 genes upregulated in the 10-dpf transcriptome of the W-hybrid were found to participate in such processes as chromosome segregation (GO:0007059) and cell cycle (GO:0007049) (Figure 6A). These genes were differentially expressed at 10 dpf, but the difference gradually waned down at 12 and 14 dpf (Figure 6B). This suggests that presumptive female gonad is characterized by an initial accelerated cell proliferation at 10 dpf.

We further examined cell proliferation by counting germ cell number and found that they started to increase in the TL laboratory strain at 10 dpf (Figure 6C). In the Nadia/TL hybrid, the germ cell numbers were indistinguishable at 8 dpf, but became higher in the W-hybrid starting from 10 dpf (Figure 6D). The Z-hybrid did not increase its germ cell number until 12 dpf, and their germ cell numbers were lower than that in the W-hybrid. In the Nadia (NA) strain, the germ cell numbers in the ZW were also higher than those in the ZZ gonads at 10 dpf (Figure 6E). We examined cell proliferation by PCNA staining (Figure 6F), which marks the S phase of the cell cycle (Schonenberger et al., 2015). There were more PCNA^+^-germ cells in the ZW than ZZ gonads at 10 dpf (Figure 6G). This suggests that the dimorphic increase of germ cells is the first sign of sex differentiation in zebrafish.

## Discussion

In this study, we analyzed transcriptomes of zebrafish germ cells at the earliest time of sex differentiation, and found female differentiation is characterized by the increased cell proliferation and advanced meiosis. We also generated mutants deficient in meiotic gene, *sycp3*, and found mutants were exclusively infertile males with a block in meiosis followed by germ cell apoptosis. We conclude that female sex differentiation is marked by increased germ cell proliferation followed by advanced meiosis.

### The Earliest Sign of Sex Differentiation Is Germ Cell Proliferation at 10 dpf

Zebrafish sex is difficult to study because their gonad differentiation is affected by environmental factors such as temperature, density, food intake, etc. To delineate mechanisms of sex differentiation, it is crucial to study the early period when the perturbation of environmental factors is at its minimum. Zebrafish reach its sexual maturity at around 3 months of age. Testes start to develop at around 1 month of age, and ovaries at around 3 weeks of age. Most studies so far focus on these time periods and ignore the sex determination window when the first signs of sexual dimorphism occur. Here we show that the earliest sign of sexual dimorphism is the difference of germ cell proliferation, which is reflected by the increased germ cell number in 10-dpf presumptive females. We show that germ cells remain dormant after they reach the gonad at 1 dpf till 8 dpf, and germ cell numbers start to increase at 10 dpf in both the TL domesticated strain and wildtype Nadia strain. It implies that the function of the putative female-dominant sex-determining gene in the W locus is probably involved in directing cell proliferation at 8–10 dpf. This hypothesis remains to be tested after the elusive sex-determining gene is identified.

We show here that the first sign of sex dimorphism in zebrafish is germ cell proliferation in females. This is in contrast with the results found in mammalian system, which is characterized by the initial changes in the testicular sertoli cells (Spiller et al., 2017). This difference may be reflected by the characteristics of the sex-determining system in the female dominant ZW/ZZ system versus the male dominant XX/XY system. Zebrafish germ cells seem to have pivotal roles as opposed to somatic cells in mammals, and zebrafish females are dominant over males in sex differentiation. Further detailed evolutionary studies will be necessary to provide more answers to the differences of the sex-determination systems.

### Novel Genes Cluster on Chromosome 17

Our 10-dpf RNA-seq reveals a cluster of genes (*FO704836.1, FO834825.3, zgc:*100951 and *zgc:113886*) in chromosome 17 upregulated in presumptive female germ cells of the W-hybrid (Figure 5C). This region has not been well sequenced, and their gene names are often overlapped in the latest version of zebrafish genome GRCz11. It is possible that there are more or fewer genes in this cluster, or some of these genes are duplicated in tandem with high sequence homology. Two of these genes, *FO704836.1, FO834825.3,* are also called *zgc:112399* in the other databases (Uniport and Alliance of Genome Resources). These genes (*FO704836.1, FO834825.3, zgc:100951*) are either paralogous genes or the same gene given different names*.* The *zgc:112399* gene is annotated to participate in cell proliferation. The *zgc:*113886 gene is annotated to encode a C2H2-type zinc finger protein, which have the DNA-binding transcription factor activity and the RNA polymerase II-specific regulation. It is possible that genes in this locus are involved in cell proliferation and consequently associated with female differentiation. But this requires more definitive characterization of the gene locus and further gain- or loss-of-function examination to exmine the functions of these genes.

### Germ Cell Numbers Determine Zebrafish Sex Differentiation Patterns

The numbers of germ cells are crucial for female gonad differentiation in zebrafish. Here we show that germ cell numbers become sex dimorphic at 10 dpf, and many genes associated with cell growth are upregulated in our transcriptomic analysis. Our results are consistent with earlier publications showing that abundance of germ cells promote female differentiation (Ye et al., 2019), and decreased germ cells lead to male development (Slanchev et al., 2005; Rodriguez-Mari et al., 2010). Germ cell proliferation is also critical for meiosis. Cdc20, the regulator of cell division, promotes female meiosis I and controls the segregation of sister chromosome in mice (Jin et al., 2010). The mutation of zebrafish *e2f5* leads to repression of cell cycle progression forming only males (Chong et al., 2018). Although the dominant factors that trigger female germ cell proliferation are still elusive, our results show that presumptive female germ cells in zebrafish advance their growth and meiosis as early as 10 dpf.

### Male Differentiation Directly or *via* Oocyte Apoptosis?

The mechanism of zebrafish testis differentiation is an issue filled with contradictory reports. Many studies find that oocyte apoptosis leads to male development after the ovary is initially formed (Uchida et al., 2002). We also find that germ cell apoptosis at 1 month of age leads to final male development in the *sycp3*-null mutants. But a direct male development pathway without going through a juvenile female phase has also been detected. Male-specific genes such as *sox9a* are expressed in a sex-dimorphic pattern at around 21 dpf (Tong et al., 2010), before the appearance of oocyte apoptosis. This points to a pathway leading to male differentiation directly from bipotential gonads. We find that female development is associated with increased germ cell proliferation and advanced meiosis at the earliest time of sexual differentiation, also pointing to an early intrinsic pathway of sex differentiation. It appears that both the initial intrinsic pathway and the later pathway caused by germ cell apoptosis can lead to final testis development. It also indicates that zebrafish sexual differentiation is very plastic and can alter in response to environmental changes.

## Data Availability

The datasets presented in this study can be found in online repositories. The names of the repository/repositories and accession number(s) can be found in the article/Supplementary Material.
