# Supplementary material for: Zebrafish Establish Female Germ Cell Identity by Advancing Cell Proliferation and Meiosis
Source: Front Cell Dev Biol. 2022 Apr 4;10:866267. doi: 10.3389/fcell.2022.866267 (PMC9013747; doi:10.3389/fcell.2022.866267)
Supplement: Supplementary file 1 [file DataSheet1.pdf]

Supplementary Materials

Zebrafish establish female germ cell identity by advancing cell proliferation and meiosis

You-Jiun Pan[1,2], Sok-Keng Tong[1], Chen-wei Hsu[1], Jui-Hsia Weng[1,3], and Bon-chu Chung[1]

[1]Institute of Molecular Biology, Academia Sinica, Taipei, Taiwan
[2]Institue of Biochemistry and Molecular Biology, National Yang Ming Chiao Tung University, Taipei, Taiwan, [3]Institute of Biological Chemistry, Academia Sinica, Taipei, Taiwan

This supplement contains two Supplementary Tables and two Supplementary Figures. The RNAseq data sets has been uploaded to the Gene Expression Ominbus database with the accession number: GSE196899 (https://www.ncbi.nlm.nih.gov/geo/query/acc.cgi?acc=GSE196899).

Supplementary Table S1. Key Resources. All key resources used in this article are listed here.

Reagent or resource	Source	Identifier	Note
**Experiment animal**			
Zebrafish/TL	Zebrafish International Resource Center	https://zfin.org/ZDB-GENO-990623-2	
Zebrafish/Nadia	From Dr. John H. Postlethwait	(Wilson, High et al. 2014)	
**Antibodies**			
Vasa<br>Rabbit polyclonal against recombinant protein encompassing a sequence within the center region of zebrafish Vasa.	GeneTex	GTX128306	IF<br>1：500
Sycp3<br>Rabbit polyclonal against synthetic peptide corresponding to Human SCP3 aa 200 to the C-terminus (C terminal) conjugated to keyhole limpet haemocyanin	Abcam	ab150292	IF<br>1：200
Rad51<br>Rabbit polyclonal	Abcam	ab137323	IF<br>1：200
PCNA<br>Mouse monoclonal Rat PCNA-protein A fusion protein	Dako	M0879	IF<br>1：500
Casp3<br>Cleaved Caspase-3 (Asp175)<br>Rabbit polyclonal	Cell Signaling Technology	Cat.#9661	IF<br>1：400
**Oligonucleotides**			
Genotype primer			
NA-F	CCGGCCCTCAAGGACCGAAA		
NA-R	GGTTGCTCAAGTGTTGGTGAGA		
sycp3 sgRNA-sequence-F	CGCAAAATACTCCTCAAAAACC		
sycp3 sgRNA-sequence-R	CAGCACACAAGGGTCAAATAAA		
sgRNA primer			
sycp3 sgRNA	gcgtaatacgactcactataCGGTGTGGTGACAGACCTGAgttttagagctagaaatagc		
Guide-constant oligo	AAAGCACCGACTCGGTGCCACTTTTTCAAGTTGATAACGGACTAGCCTTATTTTAACTTGCTATTTCTAGCTCTAAAAC		
**qPCR primer**			
EEF1al1-F	AGC AGC AGC TGA GGA GTG AT		

EEF1al1-R	TGG TTC TCT TGT CGA TTC CA		
Q-CU207311.2-F	CAGACATTTTCAGGGCTGCCA		
Q-CU207311.2-R	CAGACGACACAAACAGAGCAA		
Q-zgc:100951-F	ACATGTGATCAGTGTCTGAAGAGT		
Q-zgc:100951-R	GGGAAACCGTTTTGCTGCAT		
Q-FO704836.1-F	TGCGGCAAAAATTTTAGGGCTT		
Q-FO704836.1-R	CACACCAGTGTGGATCTTCTGA		
Q-FO834825.3-F	CACAAATGTGATCAATGCGGC		
Q-FO834825.3-R	TGTGTTCTTTAATTTGTTCCGTGTA		
QuantiFast SYBR Green RT-PCR Kit (2000)	QIAGEN	204156	
DyLight™ 488 Microscale Antibody Labeling Kit	Thermo Scientific	53025	
PCR Purification Kit	QIAquick	Cat. #28106	
MEGAshortscript T7 Transcription Kit	Thermo Scientific	AM1354	
TRUECUT CAS9 PROTEIN V2	Thermo Scientific	A36498	
Mob II	BioLabs	Cat.#R0148	For tp53 fish genotyping
DNA Screening Kit	QIAxcel	Cat. #929004	For sycp3 genotyping
Normal Goat Serum	Jason Immuno Research	Cat.#005-000-121	
Collagenase Type 4	Worthington	Cat. #LS004188	
**Transcriptome**			
SMART-seq HT Kit	Clontech (Takara Bio)	Cat. 634437 (96 rxns)	
Nextera XT DNA Library Prep Kit	Illumina	FC-131-1096 (96 rxns)	
Qubit dsDNA HS assay Kit	Invitrogen	Q32854	
Agilent High Sensitivity DNA Kit	Agilent	5067-4626	
Agilent 2100 bioanalyzer	Agilent	G2938B	
Ampure XP	Agencount	A63881	
NetSeq500 (equipment)	Illumina	NB501708	
NetSeq500 High Output kit v.2	Illumina	FC-404-2001	
NetSeq500 System Suite Version	Illumina	2.2.0	
NetSeq500 Control Software Version	Illumina	2.2.0	

**cDNA preparation from Quartz-Seq**			
10x TaKaRa Taq buffer (Mg2+)	TaKaRa	9151A	
Rnasin Plus Ribonuclease Inhibitor	Promega	N2611, N2615	
SuperScript III Rnase H- Reverse Transcriptase	Life Technologies	18080-044	
0.1 M DTT (supplied with Enzyme)	Life Technologies	18080-044	
10mM dNTP Mix	Life Technologies	18427-013	
Exonuclease I	TaKaRa	2650A	
10x Exo I bufffer (supplied with Enzyme)	TaKaRa	2650A	
Rnase H	Life Technologies	18021-014	
100 mM dATP	Life Technologies	10216-019	
Ter minal Transferase(Tdt), recombinant	Roche	3333574	
10 % NP-40	Thermo Scientific	28324	
MightyAmp DNA Polymerase Ver.2	TaKaRa	R071A, R071B	
2x MightyAmp Buffer Ver.2 (supplied with Enzyme)	TaKaRa	R071A, R071B	
DNA Clean & Concentrator -5	ZYMO RESEARCH	D4013, D4014	
Agencourt AMPure XP	BEKMAN COULTER	A63880, A63881	
**Software**			
BD FACSDiva software	Becton Dickinson biosciences	v6.2	
GraphPad Prism	https://www.graphpad.com/		
Imaris x64 9.8	https://imaris.oxinst.com/packages		
Zeiss Zen 2009 (Black edition)	https://www.zeiss.com/microscopy/int/products/microscope-software/zen.html		
CHOPCHHOP	https://chopchop.cbu.uib.no/		
Benchling	https://benchling.com/editor		

Supplementary Table S2. List of samples batches used for RNA-seq analysis. Germ cells from the W- and Z-hybrids were collected in different batches and cDNA libraries prepared using two different methods in two different sets of experiments. In first sequencing experiment, there are two batches of W and one batch of Z samples at 12- and 14 dpf, and their cDNA were performed by Quartz-seq. In second sequencing experiment, there are triplicated batches W and Z at 10 dpf, and quadruplicated batches W and Z at 12 dpf. The cDNAs of second sequencing were prepared by Smart-seq HT. The trunk tissues are used as a negative control.

#	Stage	Sample	no. of Sample Batches	Method cDNA Preparation
1st	12 dpf	W	2	Quartz-seq
		Z	1	
	14 dpf	W	2	
		Z	1	
2nd	10 dpf	W	3	Smart-seq HT
		Z	3	
	12 dpf	W	4	
		Z	4	
	Neg. Ctrl (trunk)		2	

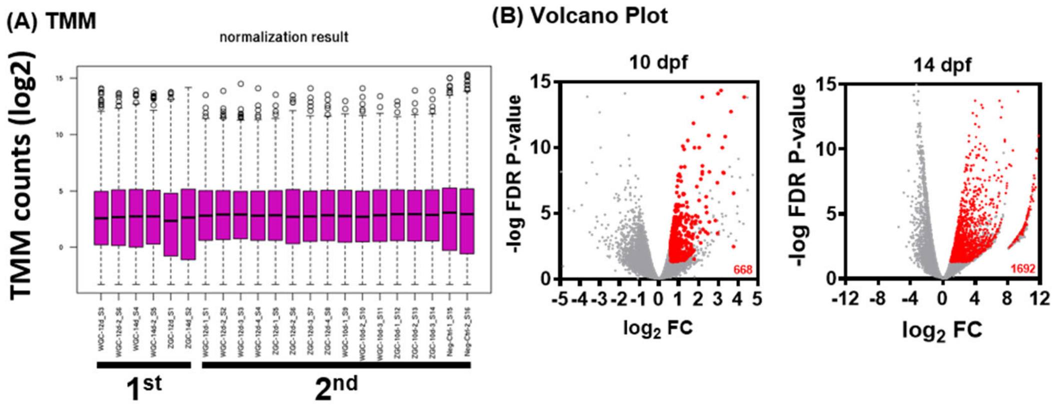

Supplementary Fig. S1. The Quality control of the transcriptomic analysis and volcano plots of 14- and 10-dpf transcriptomes

A box plot showing the normalization of RNA-seq results by the method of Trimmed Mean of the M-values (TMM) from the edgeR package R program. The X-axis shows all the sample batches used in RNA-seq. All these batches of samples have similar TMM counts, therefore can be compared. (B) The volcano plots show differentially expressed genes (DEGs) at 10- and 14-dpf. FC: fold change of gene expression in W- and Z-hybrid. FDR: false discovery rate. There are 668 genes upregulated in the W-hybrid compared to the Z-hybrid at 10 dpf and 1692 genes in 14 dpf (upregulated genes labeled in red dots, others genes labeled in gray).

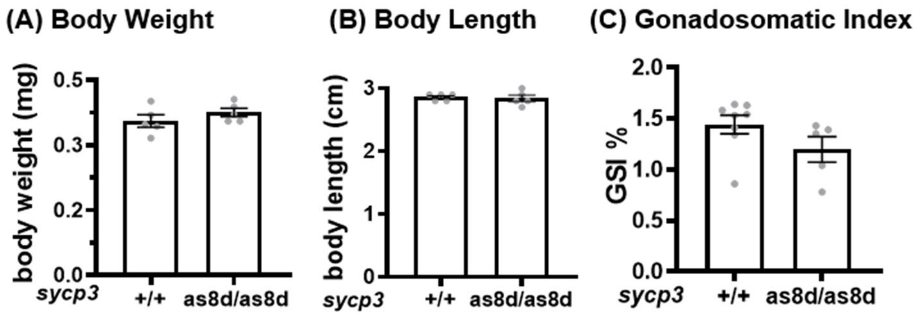

Supplementary Fig. S2. The gonad morphology of *sycp3* mutants.

(A) The body weight, (B) body length, and (C) gonadosomatic index (GSI%) of 6-month *sycp3* deficient mutants (as8d/as8d) are not changed.
